# An online experiment that presents challenges for translating rest-related gains in visual detail memory from the laboratory to naturalistic settings

**DOI:** 10.1371/journal.pone.0290811

**Published:** 2024-01-17

**Authors:** Emmi Leetham, Tamlyn Watermeyer, Michael Craig

**Affiliations:** Department of Psychology, Faculty of Health and Life Sciences, Northumbria University, Newcastle upon Tyne, United Kingdom; University of Pittsburgh School of Medicine, UNITED STATES

## Abstract

New memories are labile and consolidate over time. Contemporary findings demonstrate that, like sleep, awake quiescence supports consolidation: people remember more new memories if they experience a brief period of post-encoding quiet rest than sensory processing. Furthermore, it was recently demonstrated that the *quality* of new memories can also be enhanced significantly by awake quiescence. This phenomenon offers great applied potential, for example, in education and eyewitness testimony settings. However, the translation of rest-related gains from the laboratory to everyday life remains poorly characterised and findings are mixed. Here, we report follow-on evidence demonstrating that rest-related gains in visual detail memory may be more challenging to achieve in naturalistic than laboratory-based settings. In contrast to established laboratory findings, using an online version of an established consolidation paradigm, we observed no memory benefit of post-encoding quiescence, relative to an engaging perceptual task, in the retention of detailed visual memories as measured through a lure discrimination task. This null finding could not be explained by intentional rehearsal in those who rested or between-group differences in participants’ demographics or mental state, including fatigue and mood. Crucially, post-experimental reports indicated that those in the rest group experienced challenges in initiating and maintaining a state of quiescence, which may account for our null finding. Based on these findings, we propose three areas of focus for future work should rest-related gains in memory be translated from the lab to field: (1) to establish the specific environmental and individual conditions that are conducive and detrimental to awake consolidation, (2) to understand the barriers to initiating and maintaining a state of quiescence in naturalistic settings, and (3) to examine how knowledge of quiescence and its cognitive benefits can encourage the initiation and maintenance of states that are conductive to awake consolidation.

## Introduction

Newly encoded memories are fragile and susceptible to disruption and forgetting. To abate such forgetting, the time-dependent process of consolidation strengthens and stabilises fragile new memories [1, 2]. Consolidation is proposed to be an opportunistic process [3, 4] that is thought to be supported, at least in part, by neural reactivations of memory representations shortly following their formation [5–7]. In keeping with the opportunistic consolidation theory, “memory replay” is observed most prominently during post-encoding sleep and quiet rest periods where sensory processing and task engagement are minimised. This replay-consolidation hypothesis is supported by behavioural findings demonstrating superior memory retention following post-encoding periods of sleep [8–14] *and* awake quiescence [15–26].

The positive influence of sleep on memory consolidation is well characterized but less is known about the contribution of awake quiescence. Laboratory-based studies investigating the effects of awake quiescence on memory retention is a growing area of interest, especially given the possible therapeutic potential of quiescence to alleviate forgetting, including in those with Alzheimer’s Disease, where a pathological fault in awake consolidation is proposed to–at least partially–account for their memory dysfunction [22, 27, 28].

Growing evidence reveals that participants retain significantly more verbal and visual-associative information should they engage in a brief period of quiet rest immediately following encoding, relative to experiencing sensory processing and task engagement through an unrelated perceptual task [15–26]. Positive effects of quiescence in memory retention have been shown to remain over a time course of minutes to days [21, 29] and these effects persist even when intentional memory rehearsal during rest periods is minimised, ruling out artifacts of participants intentionally practicing the items post-encoding [30].

Apart from supporting the quantity of memories retained, recent data indicate that rest-related consolidation supports the *quality* of new visual memories [25]. Combining an awake consolidation paradigm with the Mnemonic Similarity Task [MST; 31], Craig & Dewar (2018) first presented participants with a set of photos of everyday items (targets) in sequence. Memory for these items was subsequently tested using a visual recognition test, where participants were presented another set of photos of everyday items in sequence but asked to indicate whether each item was identical to a photo presented during encoding (targets); visually similar, but subtly different, to a photo presented during encoding (lures), or a new photo not presented during encoding (foils). Two memory measures are typically extracted from the MST: a standard recognition score and a Lure Discrimination Index (LDI) score [31]. While the standard recognition measure probes participants’ gist-based representations of encoded items, the LDI measure probes the fine detail of participants’ traces for encoded items by requiring them to discriminate between encoded targets and visually similar lures [25]. Therefore, a participant with finely detailed representations of encoded photos, should show a superior ability to correctly identify subtle visual differences in lure items, and thus be able to discriminate them from targets with higher precision. Craig & Dewar’s (2018) findings showed that participants who experienced post-encoding awake acquiesce demonstrated significantly better LDI scores than those who experienced ongoing sensory processing and task engagement via a spot-the-difference game. It was theorised that this finding could be explained by superior consolidation during quiescence in the absence of interfering sensory processing.

The promotion of awake consolidation to support memory retention could have a significant impact in applied settings [32]. Such impact could be felt, for example, in education and eyewitness testimony settings, as well as broader benefits in those with and without memory disorders, where more striking effects are reported [22, 28]. Indeed, restful mindfulness and meditation states have been proposed to offer potential to support learning [33] and engagement in targeted rest periods is suggested to have great potential in sporting settings [34]. However, observation of rest-related benefits in the retention of new memories in applied settings is restricted to improved verbal memory retention in education settings [35, 36] though findings are mixed [see 37]. In attempt to bridge the gap between the laboratory and everyday life, a recent study applied an online procedure to investigate the effect of rest in the cued recall of verbal information [38]. In keeping with laboratory findings, the authors observed a significant benefit of quiescence in memory retention (i.e., memory quantity). However, no studies have attempted to translate the benefit of rest in visual detail memory (i.e., memory quality) from the laboratory to everyday settings. It is not inherent that awake quiescence should benefit detail memory in naturalistic settings. Awake quiescence requires participants to maintain a quiet restful state over several minutes. While this is achievable with relative ease under laboratory conditions, it might be challenging to engage in, and maintain, a state of quiescence in naturalistic settings with an abundance of sensory cues. Understanding the possible barriers towards achieving rest-related gains in memory quality is therefore of value.

To this end, we examined whether the benefit of awake quiescence in visual detail memory can be observed in naturalistic settings. This was achieved through the remote and online delivery of an established consolidation paradigm in younger adults [25]. In this paradigm, participants experience an incidental encoding phase for a set of photos of everyday items before experiencing a short period of awake quiescence or ongoing sensory processing and task engagement via a spot-the-difference game. In keeping with recent laboratory findings [25], should the memory benefit of quiet rest translate from the laboratory to the field, we hypothesised that individuals who experienced a post-encoding period of awake acquiesce would demonstrate superior lure discrimination for recently encoded photos, relative to individuals who experienced ongoing sensory processing and task engagement via the spot-the-difference game.

## Methods

### Ethics statement

This research was approved by the Faculty of Health and Life Sciences’ Research Ethics Committee at Northumbria University (Ref: 26608). Informed written consent was acquired from all participants following an initial study briefing and procedures adhered to the appropriate ethical principles for research in humans.

### Participants

An a priori sample size calculation was conducted using G*Power 3.1 [39]. This calculation indicated that a minimum sample of 172 participants (n = 86 per group) was required to detect a significant between-group difference in an independent samples t-test (two tailed) when considering 80% power, an alpha level of .05, and a medium effect size (d = 0.5). Rest-related gains in memory have commonly demonstrated large effect sizes (e.g., Craig & Dewar, 2018); to accommodate extraneous variables for this online study, a more conservative medium effect size was used in the power calculation. The minimum sample required (n = 172) was exceeded through the recruitment of 208 young adults (females: n = 104, males: n = 102, non-binary: n = 2; mean age = 28.16 years, SD = 4.83, age range: 18–35 years) as participants. These individuals were recruited between 3^rd^ Sept and 24^th^ November 2022 through Prolific.co’s participant panel and reimbursed at a rate of £10.11/hour. Data were accessed for analyses following completion of data collection, i.e., from 24^th^ November 2022. Participants were not identifiable through Prolific.co or the information they provided during the study. Though we had no a priori hypotheses surrounding gender, to reduce possible gender effects, balanced sampling was used to ensure equal representation of males and females; we did not exclude participants who did not identify as male or female. Inclusion criteria comprised residing in the United Kingdom, fluency in English, and no known colour blindness. Further to this, participants confirmed that they had normal or corrected-to-normal visual acuity and no known premorbid psychiatric or neurological disorders that might affect performance. Participants were allocated randomly to one of our two independent groups, which was achieved automatically through the randomisation feature through Qualtrics following the completion of our pre-experimental questionnaire. Fourteen participants were removed from our sample because they demonstrated a lack of adherence to task instructions (awake quiescence: n = 2, perceptual task: n = 2) or were more than two standard deviations from their group mean in the standard recognition or LDI measure (awake quiescence: n = 8, perceptual task: n = 2). Thus, analyses were conducted on a sample of 194 participants (awake quiescence: n = 92, perceptual task: n = 102). It is, however, worth noting that no results changed when these excluded participants were retained in our sample for analyses.

### Design

**Fig 1:**
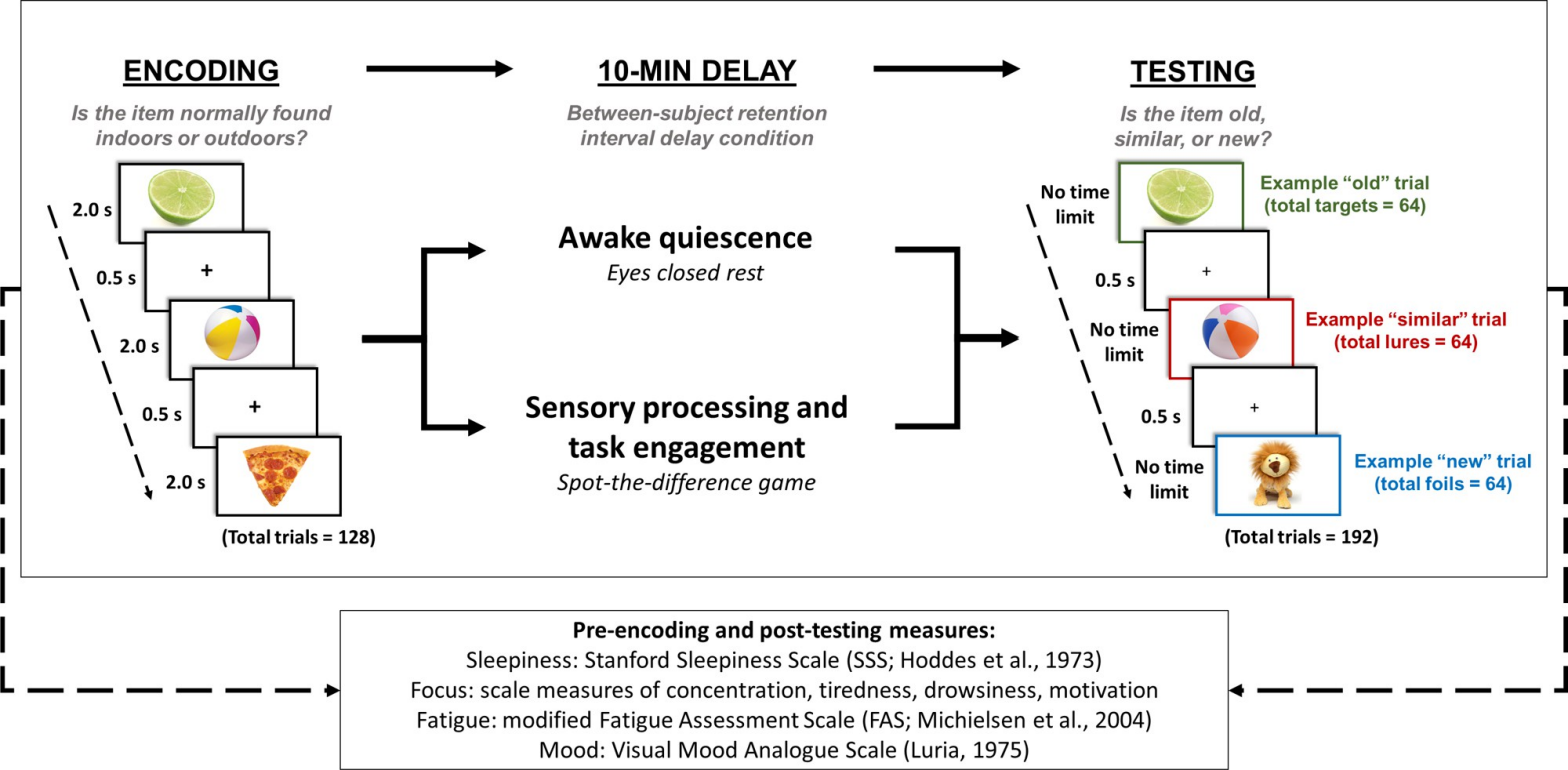
Experimental paradigm. Participants underwent three phases: (i) encoding, (ii) delay, and (iii) testing. During encoding, participants were presented with a range of 128 images of unique everyday items from the Mnemonic Similarity Task database [e.g., 31]. Participants incidentally encoded these items via the performance of a judgment making task, where they were required to respond whether a presented item would typically be found indoors or outdoors. Each item was presented for 2000ms and was followed by a 500ms inter-stimulus crosshair (+). Following encoding, participants completed one of two delay conditions: A) 10 minutes of awake quiescence (n = 92) and B) 10 minutes of an engaging perceptual task (spot-the-difference game) (n = 102). In the subsequent testing phase, participants were sequentially visually presented 192 photos of everyday items in a random order. Of these photos, 64 were old, i.e., were identical to those presented during encoding (targets), 64 were similar (visually similar items from the same semantic category) to the remaining photos presented during encoding (lures), and 64 were new to those presented during encoding. There was no limit on the time to respond during testing. Measures of sleepiness, fatigue, and mood were collected at both pre-encoding and post-testing phases. Trait rumination was also measured prior to encoding, and in the post-testing stage we retrospectively recorded state rumination and other mental activities (e.g., thoughts about the memory items) during the delay condition activities.

We employed a randomised control trial design with two independent groups to examine the effect of post-encoding behavioural state on the retention of photographs of everyday items. Crucially, in contrast to existing work, our study was delivered online and remotely. Our experimental procedure was divided into three phases: encoding, 10-minute delay phase, and testing. Our experimental manipulation (awake quiescence vs. perceptual task) occurred during the 10-minute delay phase (see Fig 1).

### Materials

Our paradigm used an adapted version of the Mnemonic Similarity Task [31, 40, 41], which was combined with an awake consolidation paradigm that has been shown to detect rest-related effects in memory [20, 23–25, 30]. The computerised task used to administer our procedure was developed using PsychoPy3 (version 2021.1.4) via a Python-coded script and delivered online via pavlovia.org. Participants completed the task on their personal laptop or PC device. Visual instructions were presented on the screen during the procedure. Stimuli (photos of everyday items) in the encoding and testing phases were presented in the centre of the screen on all occasions. Responses during encoding and testing phases were collected from participants via keyboard input. All task materials are available on the project OSF site at osf.io/usbtf/.

### Procedure

Our study employed the same experimental procedure as Craig & Dewar (2018), which was adapted for online delivery, coupled with the addition of further measures to probe participants’ subjective experiences during the post-encoding delay condition and nature of the online study. Further to this, rather than using a shortened version of the Mnemonic Similarity Task (MST), i.e., fewer stimuli, like in Craig & Dewar (2018), we employed a full stimuli set (Set C) in the current study [40]. This was done in attempt to increase test sensitivity.

#### Pre-experimental measures

A set of pre-experimental measures were implemented to uncover any extraneous variables that may skew results. This consisted of the Ruminative Response Scale [42], Stanford Sleepiness Scale [43], Modified Fatigue Assessment Scale [44], and Visual Mood Analogue Scale [45]–their aim was to assess participants’ current mental state. These measures were completed via Qualtrics before the participant was redirected to the experimental task hosted on pavlovia.org.

An adapted version of the original Ruminative Response Scale (RRS) [42] was employed to assess trait ruminative tendencies in the selected participants. Research suggests trait rumination can affect cognitive ability in some individuals, specifically, the tendency to ruminative over negative experiences and thoughts can distort an individual’s perception of reality [46]. Similarly, a ruminators’ inhibitory deficit can affect the processes of working memory and the retrieval of encoded information [46]. Therefore, to understand the effects of the current research prospective, trait rumination was controlled to understand the efficacy of memory ability fully. The RRS-short version is a self-report measure, aimed at assessing how individuals think and behave in relation to a scenario when they feel sad or depressed. The adapted version compromised of 10-items on a 4-point Likert-scale ranging from 1 –"almost never" to 4 –"almost always". Example items include ’Think "Why do I always react this way?‴ and ‘Go someplace alone to think about your feelings?’. A total score is calculated by summing all 10 items, with higher scores demonstrating a higher ruminative tendency. The adapted short version has a high correlation with the original scale (r = .90) and has a high level of internal reliability (α = .85) [47].

The Stanford Sleepiness Scale (SSS) [43] is a self-report scale designed to quantify progressive steps in sleepiness. The subjective measure aims to examine sleepiness at a current moment in time, consisting of only one item, respondents are required to select one of seven statements displayed on a 7-point Likert-scale, in relation to which best represents their level of perceived sleepiness. The scale ranges from 7 –“Feeling active, vital, alert, or wide awake” to X–“Asleep”. Early examination of the SSS has revealed scores are significantly elevated following a long period of sleep deprivation [43], similarly, research has found the scale can be utilised to predict performance on tasks related to alertness, such as reaction time, vigilance and/or memory tests [43]. In the case of the current study, sleepiness was measured as a cautious instrument to detect factors which could affect mnemonic test ability. Scores can be tested longitudinally or momentarily, at different times of day in different seasons, making it an adaptable measure for to test pre-experimental extraneous variables.

To examine participants’ ability to focus during the study procedure, they were asked to rate their current level of concentration, tiredness, drowsiness, and motivation. All items were rated on a 7-point Likert scale, where 7 related to highest intensity of the feeling, and 1 related to the near absence of the feeling.

A modified version of the Fatigue Assessment Scale (FAS) [44] was employed to evaluate symptoms of fatigue and drowsiness in participants. The self-report measure represents both mental and physical aspects of fatigue and has been validated in both male and female populations [44]. The original scale compromises of 10 items on a 5-point Likert-scale ranging from 1 –“A lot less than normal” to 5 –“A lot more than normal”, however the modified version utilised only 8 items. Participants are asked to rate the degree of their current state in relation to the items presented. Example items include “Physically, I feel exhausted” and “I feel no desire to do anything”. Items 4 and 8 were reverse scored, a total score was calculated by summing up all items which could range from a total of 8 or 40, with lower scores indicating the lowest level of fatigue, and higher scores denoting the highest. The FAS’s psychometric properties have been analysed in research to find an internal consistency of .90 [44].

A Visual Mood Analogue scale [45] was implemented to assess participants’ current mood states, this could be in the form of 8 emotions: confusion, sadness, anger, fear, energy, tiredness, happiness, or tenseness. The test is designed to quantify the measurement of mood in individuals, whereby previous methods have been too complex [48]. The scale is comprised of 8 mood items on a 100mm quasi-dimensional ordinal scale, participants are asked to indicate how they feel at this current moment in line with the presented emotion (e.g., sad, angry). This ranges from 0 –“Not at all” to 100 - “Extremely”. Total scores are generated through each individual emotion, with a high score indicating a higher level of that emotion being present in the individual and vice versa.

#### Memory test procedure

In keeping with existing research examining awake consolidation [16, 21, 25, 49] and lure discrimination [31, 40, 41], an incidental encoding phase was employed in the current study. Specifically, participants were pre-experimentally informed that they were taking part in a study investigating how humans make judgements about everyday items. They were not informed that they would be randomly allocated to one of two 10-minute delay conditions or complete a subsequent memory test for the presented items. This incidental encoding procedure is particularly relevant to awake consolidation research as it reduces the likelihood of mnemonic strategies, for example, conscious rehearsal of items during the awake quiescence delay condition, which may otherwise mask automatic and sub-conscious effects of awake consolidation [21, 30].

To this end, participants were presented with 128 photos of a range of unique everyday items from the Mnemonic Similarity Task [31, 41] and informed that they were completing a judgement making task. Each item was presented as a standalone item on a white background. Each item was presented for 2 seconds, with an inter-stimuli crosshair (+) appearing in the centre of the screen for 0.5 seconds (total duration of encoding phase = 320 seconds). Thus, all participants received identical treatment and exposure to stimuli during encoding. When presented with an item, participants were required to judge whether they believed the item would typically be found indoors or outdoors. For example, if presented a photo of a sofa, this item would typically be found indoors, but if presented a photo of a tree, this item would typically be found outdoors. Participants input responses via the ‘z’ (indoors) and ‘m’ (outdoors) keys on the keyboard and were instructed to respond as fast as possible; they were able to respond within the 2 second stimulus presentation window. Participants were instructed that some items may be ambiguous in their typical location (i.e., may be found indoors and outdoors), but that they should respond as quickly as possible and respond with their first instinct.

During the delay phase, participants experienced one of two delay conditions, where they either (i) rested wakefully for 10 minutes between the encoding and testing phases (i.e., quiet delay) (N = 92) or (ii) performed an unrelated perceptual task (a spot-the-difference game) for 10 minutes between the encoding and testing phases (i.e., perceptual task delay) (N = 102). Participants assigned to the *quiet rest delay* condition were asked to sit quietly and rest with their eyes closed in a room of their choosing with consideration of little to no distractions being present during this time. This was to ensure that participants’ rooms were free of any rich visual and/or audible sensory cues which could disrupt the process of consolidation. Throughout the duration of the rest delay, participants were presented a black screen, which was overlaid with a small white cross in the centre of the screen to demonstrate to the participant that the experimental task was still active. Five seconds prior to the end of the rest delay, participants were presented a 1-second ‘A’ tone to indicate that they had reached the end of the rest period.

Participants assigned to the *perceptual task* delay were asked to play a visual spot the difference game [21, 25]. Participants performed a total of 40 spot-the-difference trials, each 15 second in duration. A trial consisted of the presentation of a pair of real-world photos on the computer screen (see Fig 1 for examples). Photos were either (i) identical other than for two discrete differences (total items = 20) or (ii) identical and contained no differences (total items = 20). The order of identical and subtly different photos was randomised. Participants were informed that some photos would be identical and others would contain two subtle differences, where their task was to search for any differences, and to press the spacebar each time a difference was discovered. There was no cap on the number of times that the participant could respond and if two differences were found before the end of a trial, participants were asked to continue looking at the photos until a new trial started. All spot-the-difference task photos comprised real-world scenes, including landscapes, objects, people, and animals in various contexts. Attempts were made to minimise possible overlap between the contents of the spot-the-difference photos and the MST photos of everyday items though some relatedness remained, for example, between a photo of a plant (MST stimulus) and an outdoor scene containing vegetation (spot-the-difference game stimulus). Instructions for the quiet rest and perceptual task delay conditions were presented on screen immediately following the end of the incidental encoding phase and immediately prior to the allocated 10-minute delay condition.

Following the delay phase, all participants performed an online version of the Mnemonic Similarity Task (MST) [31, 40, 41], which probed the fine detail of memories for stimuli presented during the encoding phase. In this test, participants were sequentially visually presented 192 photos of everyday items in a random order. Of these photos, 64 were *old*, i.e., were identical to those presented during encoding (targets), 64 were *similar* (visually similar items from the same semantic category) to the remaining photos presented during encoding (lures), and 64 were *new* to those presented during encoding (foils)—see Fig 1 for examples. Participants were informed that, like in the encoding phase, they would be visually presented a set of photos of everyday items in the centre of the computer screen. However, on this occasion, rather than performing an “indoor/outdoor” judgment-making task, their memory for the earlier presented items would be probed. They were instructed that presented items would be either (i) visually identical to those presented earlier (old targets), (ii) visually similar to those presented earlier (similar lures), or (ii) brand new and not presented earlier (new foils). Using the computer keyboard, they were asked to provide an ‘old’ (visually identical target item; ‘z’ key), ‘similar’ (visually similar lure item; ‘v’ key), or ‘new’ (new foil item; ‘m’ key) response. Participants were informed that there was no time limit to respond, and they should respond as accurately as possible. Onscreen instructions regarding which keys corresponded to which response (e.g., ‘z’ = old) were always shown during encoding and testing. The order of test items was randomised to reduce the possibility of order presentation effects.

#### Post-experimental measures

At the end of the testing phase, participants were redirected from the experimental task to Qualtrics, where they completed a set of post-experimental measures to assess their current mental state, including reporting their current mood, levels of sleepiness, and ruminative tendencies. Both the quiescence and perceptual delay groups were provided the same questionnaires; question wording was in accordance with the delay experienced. The presented measures comprised the Modified Resting-State Questionnaire [50], Adapted Brief State Rumination Inventory [51], Stanford Sleepiness Scale [43], Modified Fatigue Assessment Scale [44], a scale-based measure of participants ability to focus, and the Mood Visual Analogue Scale [45]. Participants were also asked to report whether they engaged in mnemonic strategies and thought about the studied items during their allocated delay condition. Measures of sleepiness, focus, fatigue, and mood were delivered as described in the pre-experimental measures section above. The other measures were delivered as follows.

To obtain insights into participants mental activities pertaining to encoded items during their allocated delay condition, participants were first asked whether they engaged in any strategies to help them remember the encoded items. Further to this, using an existing scale from related research [17], participants were asked to rate the regularity that they (i) thought about, (ii) imagined, and (iii) remembered the encoded stimuli. For each aspect, they were asked to provide a rating on a 5-point Likert scale, where 1 = not at all and 5 = constant.

A modified version of the Resting State Questionnaire (ReSQ) [50] was employed to assess participants’ inner emotional experience following a resting state. The questionnaire was devised into 5 types of question in accordance with the quiet delay phase. Example items included were “Did you keep your eyes closed for the full duration of the break?”, this was to ensure participants followed initial instructions to achieve a wakeful state of resting. Secondly, “Did you stay awake during the full rest break?” and “Did you experience any recurrent thoughts or themes when resting?” assessed whether participants rested to their full potential without the influence of internal distractions. This type of structure following a resting state reduces inter-experimenter variability and permits application to a large sample size [50]. Ultimately, responses from the questionnaire allowed researchers to assess the quality of rest experienced.

The adapted Brief State Rumination Inventory (BSRI) [51] is a self-report measure designed to evaluate state rumination. The questionnaire requires the participant to focus on a current scenario to assess the degree of how persistent and repetitive thoughts appear. The scale demonstrates the ability to be highly sensitive toward short-lived changes in the intensity of rumination [51], thus appealing to the current study where transient increases and decreases of rumination are thought to affect the validity of results. The adapted version compromises of 13 items on a 100mm visual analogue scale (VAS) ranging from 0 –“completely disagree” to 100 –“completely agree”. The survey asks participants to respond in accordance with their emotional thoughts during the 10 minutes they rested quietly, or during the 10-minute spot the difference task. Example items include “I reflect about my mood” and “I rehearsed in my mind recent things I’ve said or done”. Higher scores indicate a higher level of state rumination.

### Scoring

Performance in the MST was scored as in previous work [25, 31, 40, 41]. Firstly, the total number of *old*, *similar*, and *new* responses for targets, lures, and foil items was extracted and converted to proportion scores (each score /64). From these proportion scores, two key memory measures were calculated: (i) a standard recognition score, and a Lure Discrimination Index (LDI) score [31]. The standard recognition score is calculated as the proportion of “old” responses to targets minus the proportion of “old” responses to foils, and thus reflects a person’s ability to correctly endorse targets, while rejecting foils. The LDI score is calculated as the proportion of “similar” responses to lures minus the proportion of “similar” responses to foils, and thus reflects a person’s ability to discriminate lures from targets, while controlling for any response biases [31, 52]. Finally, we extracted the time it took participants to respond during encoding and testing to check for potential group differences in encoding and retrieval.

### Statistical analyses

Analyses were performed using SPSS Statistics 28 (copyright IBM Corp., NY, USA), with the alpha level set to .05. Independent samples t-tests were conducted to examine possible differences between our independent delay condition groups (awake quiescence vs perceptual task) in their demographic and background details (e.g., age, visual acuity), state and trait measures (e.g., sleepiness, mood) encoding performance, memory scores (standard recognition, LDI), and task response times. Bonferroni-corrected alpha levels were applied (p = 0.05 / # comparisons) to correct for multiple within-family comparisons. All study data are available on the project OSF site at osf.io/usbtf/.

## Results

### Demographic and background measures

Groups were matched in their age (awake quiescence: M = 28.33 years, SD = 4.67 years, perceptual task: 28.12 years, SD = 5.02 years; t(192) = 0.30, p = 0.77, d = 0.04) and gender distribution (awake quiescence: female n = 43, male n = 49; perceptual task: female n = 55, male n = 45, non-binary n = 2; *X*^*2*^ (2, N = 194) = 3.13, p = 0.21).

### Visual acuity

All participants reported normal or corrected-to-normal vision, for example, through wearing contact lenses or glasses. To qualify participants’ visual acuity, we pre-experimentally examined their responses to the Quality of Vision (QoV) questionnaire [53]. Summed scores across three subscales (frequency, severity, bothersome; max score = 40 in each) demonstrated no substantive difficulties in vision across our sample in the frequency (M = 1.98, SD = 2.70) or severity (M = 1.46, SD = 2.16) of ocular problems (e.g., glare or difficulties focusing), and such problems were not bothersome (M = 0.94, SD = 2.08). Participants allocated to each delay condition group were matched in their self-reported vision quality (frequency: t(192) = 0.15, p = 0.88, d = 0.02; severity: t(192) = -0.08, p = 0.47, d = -0.01; bothersome: t(192) = 0.60, p = 0.55, d = 0.09).

### Trait rumination

Probing of trait rumination through an adapted version of the Ruminative Response Scale [54], revealed that groups were matched in their typical rumination activity (awake quiescence: M = 21.22, SD = 20.04; perceptual task: M = 20.04, SD = 6.17; t(192) = 1.37, p = 0.17, d = 0.20).

### Pre-experimental measures

#### Sleepiness

Stanford Sleepiness Scale [43] scores revealed that our awake quiescence (M = 2.16, SD = 0.83) and perceptual task (M = 2.19, SD = 0.88) groups reported feeling sufficiently alert to complete our study. Mean scores in both groups reflected a response of “functioning at high levels, but not at peak; able to concentrate” most closely. Sleepiness ratings did not differ between groups (t(192) = -0.19, p = 0.85, d = -0.03).

#### Focus

Participants rated their concentration, tiredness, drowsiness, and motivation on 7-point Likert scales, where 1 corresponded to *near absence* and 7 corresponded to *high intensity*. Comparison of these data indicated that groups were matched in these traits; no significant between-group differences were observed in concentration (awake quiescence: M = 5.37, SD = 1.04; perceptual task: M = 5.57, SD = 0.92; t(192) = -1.42, p = 0.16, d = -0.20), tiredness (awake quiescence: M = 2.72, SD = 1.44; perceptual task: M = 2.55, SD = 1.40; t(192) = 0.83, p = 0.41, d = 0.12), drowsiness (awake quiescence: M = 1.77, SD = 1.06; perceptual task: M = 1.86, SD = 1.26; t(192) = -0.54, p = 0.59, d = 0.20), motivation (awake quiescence: M = 4.75, SD = 1.36; perceptual task: M = 4.88, SD = 1.38; t(192) = -0.67, p = 0.50, d = 0.19).

#### Fatigue

A modified version of the Fatigue Assessment Scale [44] was used to probe pre-experimental fatigue further. Across eight statements, for example, *“I feel fatigued”*, participants rated their current state on a 5-point Likert scale, where 1 = *a lot less than normal*, 3 = *as per normal*, and 5 = *a lot more than normal*. Table 1 reports mean scores across all eight statements. A comparison of scores revealed that the awake quiescence and perceptual task groups were matched in their self-reported fatigue (all p > 0.17; see Table 1) and no significance values met a Bonferroni-corrected alpha value of p = .006 (p = .05 / 8 comparisons). Scores also reveal that participants were not experiencing levels of fatigue that would be expected to influence task performance negatively.

**Table 1:** Modified fatigue assessment scale.

Statement	Awake quiescence	Perceptual task	t-test
*I feel fatigued*	2.39 (0.91)	2.41 (0.94)	t(192) = -0.15, p = 0.88, d = -0.02
*I feel tired*	2.43 (0.92)	2.37 (0.97)	t(192) = 0.46, p = 0.65, d = 0.07
*I feel energetic*	3.04 (0.88)	3.07 (0.76)	t(192) = 0.38, p = 0.83, d = -0.03
*Physically*, *I feel exhausted*	2.15 (1.05)	2.08 (0.93)	t(192) = 0.22, p = 0.60, d = 0.08
*I can think clearly*	3.47 (0.82)	3.47 (0.75)	t(192) = 0.63, p = 0.98, d = -0.00
*I feel no desire to do anything*	2.21 (1.08)	2.00 (1.01)	t(192) = 0.40, p = 0.17, d = 0.20
*Mentally*, *I feel exhausted*	2.17 (1.07)	2.06 (1.04)	t(192) = 0.63, p = 0.45, d = 0.11
*I can concentrate*	3.68 (0.84)	3.66 (0.72)	t(192) = 0.14, p = 0.80, d = 0.04

Mean scores and standard deviations (in parentheses) for the awake quiescence and perceptual task groups are shown for each of the eight statements presented to participants. Outcomes of independent t-tests comparing data between our experimental groups are also reported. No significance values met a Bonferroni-corrected alpha value of p = .006 (p = .05 / 8 comparisons).

#### Mood

Participants rated their mood using a visual analogue scale [45] across eight domains (see Table 2) on a 0–100 scale, where 0 corresponded to the complete absence of that feeling (*“not at all”*) and 100 related to the extreme experiencing of the feeling. Low levels of feeling afraid, confused, sad and angry were observed. In addition, moderate feelings of tiredness and tension were reported, along with more intense experiences of energetic and happy feelings (see Table 2). Comparison of mean scores between groups revealed no significant differences (all p > 0.05; see Table 2) and no significance values met a Bonferroni-corrected alpha value of p = .006 (p = .05 / 8 comparisons), which indicates that groups were matched in their mood when entering the study.

**Table 2:** Visual analogue mood scale.

Measure	Awake quiescence	Perceptual task	t-test
*Afraid*	9.33 (17.56)	6.50 (13.80)	t(192) = 1.25, p = 0.21, d = 0.18
*Confused*	7.73 (13.80)	6.49 (12.22)	t(192) = 0.47, p = 0.51, d = 0.10
*Sad*	16.68 (23.02)	12.68 (20.20)	t(192) = 0.11, p = 0.20, d = 0.19
*Angry*	10.75 (19.61)	6.12 (13.48)	t(192) = 1.93, p = 0.06, d = 0.28
*Energetic*	52.59 (23.78)	45.83 (27.70)	t(192) = 1.81, p = 0.07, d = 0.26
*Tired*	27.61 (24.79)	21.92 (20.81)	t(192) = 1.74, p = 0.08, d = 0.25
*Happy*	54.36 (24.64)	52.67 (24.74)	t(192) = 0.48, p = 0.63, d = 0.07
*Tense*	20.97 (24.82)	14.88 (20.28)	t(192) = 1.88, p = 0.06, d = 0.27

Mean scores and standard deviations (in parentheses) for the awake quiescence and perceptual task groups are shown for each of the eight feelings presented to participants. Outcomes of independent t-tests comparing data between our experimental groups are also reported. No significance values met a Bonferroni-corrected alpha value of p = .006 (p = .05 / 8 comparisons).

### Memory test performance

#### Encoding

Groups were matched in their performance of the encoding phase, which comprised a judgement-making task that required participants to note whether the item shown in a photo would typically be found indoors or outdoors. There was no significant difference between groups in the number of encoding trials (total n = 128) that participants responded to (awake quiescence: M = 125.63, SD = 3.15, perceptual task: M = 125.76, SD = 2.60; t(192) = -0.33, p = 0.75, d = -0.05). Similarly, participants in both groups were matched in the mean number of “indoor” responses (awake quiescence: M = 81.27, SD = 9.89, perceptual task: M = 80.63, SD = 9.63; t(192) = -0.46, p = 0.65, d = 0.07) and “outdoor” responses (awake quiescence: M = 44.36, SD = 9.86, perceptual task: M = 45.14, SD = 9.50; t(192) = -0.56, p = 058, d = -0.08) judgements. Responses times were also matched between groups (awake quiescence: M = 0.97 s, SD = 0.16 s, perceptual task: M = 0.97 s, SD = 0.14 s; t(192) = -0.07, p = 0.95, d = 0.01). These data demonstrate that participants engaged sufficiently with the requirements of the encoding task and that this engagement was matched across groups.

#### 10-minute delay condition

For individuals assigned to the perceptual task group, data from the spot-the-difference game [21] completed during the 10-minute retention interval demonstrated engagement with the task requirements. The mean number of correct trials was 33.20 (/40) (SD = 5.22). When examining trials that did and did not contain subtle differences in the presented photos, our data reveal that participants responded correctly to 17.10 (/20) *same* trials (i.e., they did not respond when no difference existed) and 16.10 (/20) *different* trials (i.e., they pressed the spacebar at least once to note that a difference was discovered). The total number of spacebar responses to different trials ranged from 7 to 39 (M = 17.62, SD = 4.21), where the mean number of spacebar responses per different spot-the-difference trial ranged from 0.9 to 2.00 (M = 1.10, SD = 0.18).

#### Testing

**Fig 2:**
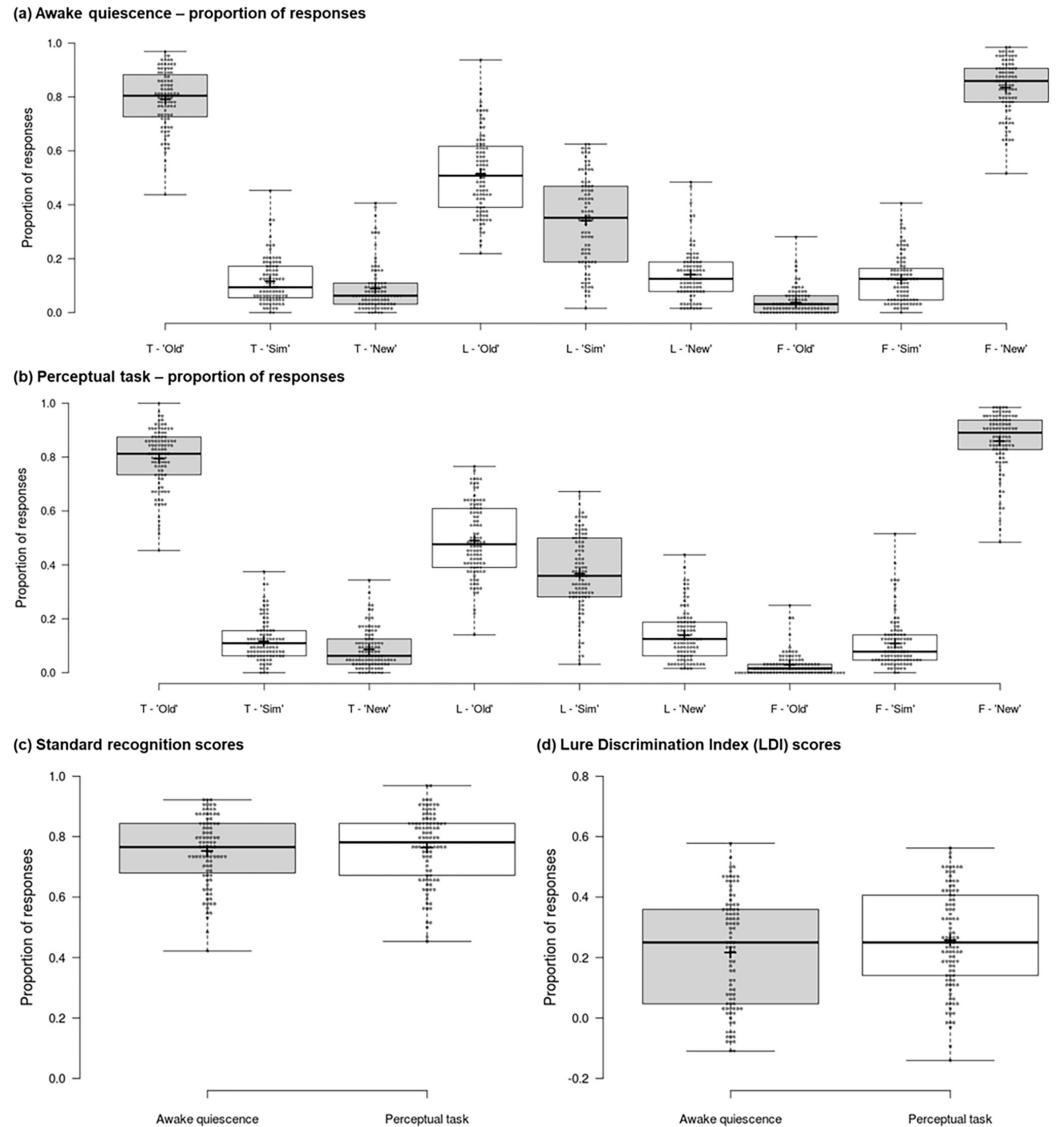
Memory test scores. Mnemonic Similarity Task (MST) [31, 40] response data for the awake quiescence (n = 92) and perceptual task (n = 102) groups. The proportion of ‘old’, ‘similar’, and ‘new’ responses to target (‘T’), lure (‘L’), and foil (‘F’) items are shown for the awake quiescence (a) and perceptual task (b) groups. Standard recognition scores are also shown (c)–these scores are calculated as the proportion of “old” responses to target items minus the proportion of “old” responses to foils, and thus reflects a person’s ability to correctly endorse old targets, while rejecting new foils. Lure Discrimination Index (LDI) scores for the two groups are also shown (d)—this measure is calculated as the proportion of “similar” responses to lures minus the proportion of “similar” responses to foils and thus reflects a person’s ability to discriminate similar lures from old targets, while controlling for any response biases. No significant between-group differences were observed in standard recognition (p = .437) or LDI (p = .104) scores. Similarly, no significant differences were observed in the proportion of ‘old’, ‘similar’, and ‘new’ responses to target, lure, and foil items (all p > .103). Solid centre lines show the medians; box limits indicate the 25th and 75th percentiles as determined by R software; whiskers extend to the minimum and maximum values; crosses represent sample means; data points are plotted as open circles.

Fig 2 shows scores in our dependent measures as a function of group. Crucially, we observed no significant group differences in the standard recognition measure (t(192) = -0.78, p = 0.44, d = -0.11; Fig 2C) or Lure Discrimination Index (LDI; t(192) = -1.64, p = 0.10, d = -0.24; Fig 2D) scores of our memory test. These null findings were reinforced by comparing the proportion of *‘old’*, *‘similar’*, and *‘new’* responses to target, lure, and foil items. Specifically, no significant between-group differences (all p > 0.10; Fig 2A and 2B) were observed across nine independent t-tests, one for each item-response combination (e.g., *“old”* responses to targets, *“similar”* responses to foils).

Mean response times across testing items did not differ significantly between groups (awake quiescence: 1.74 s, SD = 0.31 s, perceptual task: 1.78 s, SD = 0.37 s; t(192) = -0.74, p = 0.46, d = -0.11). This was also true when examining response times for target (awake quiescence: 1.67 s, SD = 0.33 s, perceptual task: 1.72 s, SD = 0.52 s; t(192) = -0.89, p = 0.37, d = -0.13), lure (awake quiescence: 1.81 s, SD = 0.39 s, perceptual task: 1.87 s, SD = 0.49 s; t(192) = -0.97, p = 0.36, d = -0.140), and foil (awake quiescence: 1.76 s, SD = 0.30 s, perceptual task: 1.75 s, SD = 0.38 s; t(192) = 0.15, p = 0.88, d = 0.02) items. Paired t-tests revealed that participants responded significantly faster to targets (M = 1.70 s, SD = 0.44 s) than lures (M = 1.84 s, SD = 0.44 s; t(193) = -4.83, p < 0.001, d = -3.47) and foils (M = 1.76 s, SD = 0.34 s; t(193) = -2.02, p = 0.044, d = -0.15). Participants were also significantly slower to respond to lures than foils (t(193) = 3.43, p < 0.001, d = 0.39).

### Post-experimental measures

#### Mnemonic strategies

Participants rated the regularity they thought about, imagined, and remembered the encoded stimuli during their allocated delay condition on a 5-point Likert scale, where 1 = not at all and 5 = constant. Comparison of these data revealed no significant difference in the regularity of thoughts about the encoded items (awake quiescence: M = 2.48, SD = 1.01; perceptual task: M = 2.80, SD = 1.33; t(192) = -1.91, p = 0.06, d = -0.27). We did, however, find that participants allocated to the perceptual task group reported imagining and remembering the stimuli to a significantly greater level than individuals in the awake quiescence group (awake quiescence: M = 2.64, SD = 1.26; perceptual task: M = 2.26, SD = 0.97; t(192) = -2.32, p = 0.02, d = -0.33). Similarly, participants in the perceptual task delay reported greater remembering of the encoded items (awake quiescence: M = 2.17, SD = 1.16; perceptual task: M = 3.00, SD = 1.53; t(192) = -4.20, p < 0.001, d = -0.60). When a Bonferroni-corrected alpha level of 0.017 was applied (p = 0.05 / 3 comparisons), only the difference in the rate of remembering the stimuli remained significant.

When asked if they employed any form of strategy to retain items presented during the encoding phase of our memory test procedure, one participant in the perceptual task group (/102, 0.98%) reported an active strategy, which comprised searching for items in the spot-the-difference game that appeared in the encoding phase. All other participants in this group reported no strategy and/or focusing solely on the requirements of the perceptual task delay. In contrast, fourteen participants in the awake quiescence group (/92, 15.22%) reported using a strategy. These strategies included: verbal rehearsal of items (n = 4), recalling the items visually (n = 6), and recalling perceptual features of the items (n = 4).

#### Delay condition thoughts and activities

In the awake quiescence delay, n = 84 participants (/92, 91.30%) reported staying awake throughout the awake quiescence delay, four participants (4/92, 4.35%) were unsure if they remained awake, and four participants (4/92, 4.35%) reported sleeping during the delay, where durations ranged from 3 to 10 minutes. Fifty-two (/92, 56.53%) participants allocated to the awake quiescence delay reported not keeping their eyes closed for the delay. In most cases, participants only temporarily opened their eyes, for example, “for a few seconds”. However, 14 (/92, 15.22%) participants reported keeping their eyes open for more extended durations between 1–5 minutes. Eleven participants (/92, 11.96%) reported facing obstacles when resting. Obstacles included boredom (n = 1), struggling to “switch off” (n = 2), sensory stimulation including a ticking sound (n = 5), becoming distracted by own thoughts (n = 1), and inexperience in maintaining a restful state (n = 2).

In the perceptual task delay, all participants (n = 102, 100%) reported remaining awake and keeping their eyes open (other than for brief moments, e.g., blinking) throughout the spot-the-difference game. Two participants (/102, 1.96%) reported environmental noise causing mild distraction when asked whether they faced any obstacles in completing the spot-the-difference game. No other challenges were reported though 15 participants (/102, 14.71%) did note task-related consequences, including finding the game challenging and feeling pressured mildly by the fixed duration of spot-the-difference game trials.

In probing the form of participants’ thoughts during their allocated delay condition, both groups mainly reported commonly engaging in thoughts that were dominated by mental imagery (awake quiescence: 43.26%, SD = 31.64%; perceptual task: 49.17%, SD = 30.20%), followed by inner language (awake quiescence: 29.67%, SD = 25.96%; perceptual task: 23.31%, SD = 25.37%) and somatosensory awareness (awake quiescence: 16.28%, SD = 20.36%; perceptual task: 17.39%, SD = 22.02%). In addition, thoughts surrounding inner musical experience (awake quiescence: 6.68%, SD = 13.06%; perceptual task: 5.80%, SD = 11.50%) and the mental manipulation of numbers (awake quiescence: 4.10%, SD = 8.77%; perceptual task: 4.32%, SD = 8.76%) also contributed to participants mental activities during their allocated delay condition. Nevertheless, comparing mean values for each form of thought between groups revealed no significant differences (all p > 0.09). This indicates that when retrospectively considering the form of thoughts that occurred during their allocated delay, the form of thoughts in those in the awake quiescence and perceptual task groups were comparable. However, the above data provide no indication whether the content of such thoughts between groups was similar.

When asked to rate how restful their delay condition was on a 7-point Likert scale, where 1 = *not restful at all* and 7 = *very restful*, we observed no significant between-group difference in ratings (awake quiescence: M = 4.13, SD = 1.61; perceptual task: M = 3.83, SD = 1.46; t(192) = 1.13, p = 0.26, d = 0.16).

Finally, to probe ruminative thoughts during their allocated post-encoding delay condition, participants completed an adapted version of the Brief State Rumination Inventory (BSRI) Questionnaire [51]. We observed no significant difference in BSRI scores between the awake quiescence (M = 250.89, SD = 410.96) and perceptual task (M = 355.54, SD = 380.18) groups (t(192) = -0.082, p = 0.94, d = -0.01). Pearson correlations revealed no significant relationship between BSRI scores and standard recognition or LDI scores in the awake quiescence or perceptual task groups (all p > 0.12).

### State measures

#### Sleepiness

Stanford Sleepiness Scale [43] scores revealed that our awake quiescence (M = 4.42, SD = 3.12) and perceptual task (M = 4.04, SD = 2.89) groups were matched in their feeling of sleepiness (t(192) = -0.89, p = 0.37, d = .13). Group means corresponded broadly to a “somewhat foggy, let down” response.

When pre- and post-experimental measures of sleepiness were entered into a RM ANOVA, we observed a significant main effect of time (F(1,192) = 87.20, p < 0.001, ηp^2^ = 0.31) because, unsurprisingly, participants felt sleepier later in our procedure. There was, however, no main effect of group (F(1,192) = 0.63, p = 0.43, ηp^2^ = 0.00) and no interaction between time and group (F(1,192) = 0.86, p = 0.36, ηp^2^ = 0.00). Thus, the increase in sleepiness was comparable across groups.

#### Focus

Participants again rated their concentration, tiredness, drowsiness, and motivation on 7-point Likert scales, where 1 corresponded to *near absence* and 7 corresponded to *high intensity*. As for pre-experimental measures, comparison of these post-experimental data indicated that groups were matched in these traits; no significant between group differences were observed in concentration (awake quiescence: M = 4.86, SD = 1.37; perceptual task: M = 4.78, SD = 1.31; t(192) = 0.39, p = 0.70, d = 0.06), tiredness (awake quiescence: M = 2.89, SD = 1.56; perceptual task: M = 3.01, SD = 1.47; t(192) = -0.54, p = 0.59, d = -0.08), drowsiness (awake quiescence: M = 2.35, SD = 1.57; perceptual task: M = 1.86, SD = 1.26; t(192) = 0.87 p = 0.39, d = 0.12), motivation (awake quiescence: M = 4.14, SD = 1.52; perceptual task: M = 4.22, SD = 1.35; t(192) = -0.36, p = 0.72, d = -0.05). When pre- and post-experimental scores were entered into a RM ANOVA, no significant main effect of time of test (F(1,192) = 3.66, p = 0.06, ηp^2^ = 0.02), group (F(1,192) = 0.17, p = 0.68, ηp^2^ = 0.00), or interaction between time of test and group (F(1,192) = 0.38, p = 0.54, ηp^2^ = 0.00) were observed.

#### Fatigue

Across eight statements from the Fatigue Assessment Scale [44], participants rated their current state on a 5-point Likert scale, where 1 = *a lot less than normal*, 3 = *as per normal*, and 5 = *a lot more than normal*. Table 3 reports mean scores across all eight statements. Comparison of scores revealed that the awake quiescence and perceptual task groups were matched in their self-reported fatigue (all p > 0.25; see Table 3) and no significance values met a Bonferroni-corrected alpha value of p = .006 (p = .05 / 8 comparisons). As during pre-experimental measurement, mean scores reflect a normal level of fatigue thar would not be expected to negatively affect performance. When pre- and post-experimental fatigue scores were entered into a RM ANOVA, no significant main effect of time of test (F(1,192) = 0.18, p = 0.67, ηp^2^ = 0.00), group (F(1,192) = 0.36, p = 0.55, ηp^2^ = 0.00), or interaction between time of test and group (F(1,192) = 0.41, p = 0.52, ηp^2^ = 0.00) were observed.

**Table 3:** Modified fatigue assessment scale.

Statement	Awake quiescence	Perceptual task	t-test
*I feel fatigued*	2.32 (1.09)	2.55 (0.95)	t(192) = -0.92, p = 0.36, d = -0.13
*I feel tired*	2.53 (1.12)	2.68 (1.02)	t(192) = -0.94, p = 0.25, d = -0.14
*I feel energetic*	2.96 (0.91)	2.93 (0.86)	t(192) = 0.20, p = 0.84, d = 0.03
*Physically*, *I feel exhausted*	2.20 (0.95)	2.11 (0.97)	t(192) = 0.63, p = 0.53, d = 0.09
*I can think clearly*	3.38 (0.81)	3.40 (0.76)	t(192) = -0.19, p = 0.85, d = -0.03
*I feel no desire to do anything*	2.25 (1.12)	2.11 (0.98)	t(192) = 0.94, p = 0.35, d = 0.14
*Mentally*, *I feel exhausted*	2.33 (1.11)	2.21 (1.05)	t(192) = 0.78, p = 0.44, d = 0.11
*I can concentrate*	3.41 (0.77)	3.50 (0.82)	t(192) = -0.76, p = 0.40, d = -0.11

Mean scores and standard deviations (in parentheses) for the awake quiescence and perceptual task groups are shown for each of the eight statements presented to participants. Outcomes of independent t-tests comparing data between our experimental groups are also reported. No significance values met a Bonferroni-corrected alpha value of p = .006 (p = .05 / 8 comparisons).

#### Mood

As done so pre-experimentally, participants rated their mood using a visual analogue scale [45] across eight domains (see Table 2) on 0–100 scale, where 0 corresponded to the complete absence of that feeling (*“not at all”*) and 100 related to the extreme experiencing of the feeling. Low levels of feeling afraid, confused, sad and angry were observed. Moderate feelings of tiredness and tension were reported along with the more intense experiencing of energetic and happy feelings (see Table 4). Comparison of mean scores between groups revealed no significant differences (all p > 0.11; see Table 4) and no significance values met a Bonferroni-corrected alpha value of p = .006 (p = .05 / 8 comparisons), which indicates that groups were matched in their mood when entering the study. When pre- and post-experimental mood scores were entered into a RM ANOVA, we observed a significant main effect of time of test (F(1,192) = 11.24, p < 0.001, ηp^2^ = 0.06). This was because participants reported a significant reduction in feelings of sadness, happiness, and tension, and feeling significantly more energetic (all p < 0.05). No significant main effect of group was observed (F(1,192) = 3.21, p = 0.08, ηp^2^ = 0.02) and there was no interaction between time of test and group (F(1,192) = 5.94, p = 0.06, ηp^2^ = 0.03), indicating that the reported changes over time were comparable between groups.

**Table 4:** Visual analogue mood scale.

Measure	Awake quiescence	Perceptual task	t-test
*Afraid*	5.25 (10.26)	4.97 (9.06)	t(192) = 0.20, p = 0.84, d = 0.03
*Confused*	7.25 (13.00)	5.85 (10.58)	t(192) = 0.82, p = 0.41, d = 0.12
*Sad*	8.26 (15.16)	10.31 (18.03)	t(192) = -0.85, p = 0.39, d = -0.12
*Angry*	6.10 (11.83)	5.54 (9.89)	t(192) = 0.36, p = 0.72, d = 0.05
*Energetic*	44.32 (27.71)	41.13 (28.96)	t(192) = 0.78, p = 0.44, d = 0.11
*Tired*	25.18 (24.57)	30.95 (25.82)	t(192) = -1.59, p = 0.11, d = -0.23
*Happy*	48.93 (26.89)	47.23 (26.89)	t(192) = 0.44, p = 0.66, d = 0.06
*Tense*	11.12 (16.21)	14.21 (17.76)	t(192) = -1.26, p = 0.21, d = -0.18

Mean scores and standard deviations (in parentheses) for the awake quiescence and perceptual task groups are shown for each of the eight feelings presented to participants. Outcomes of independent t-tests comparing data between our experimental groups are also reported. No significance values met a Bonferroni-corrected alpha value of p = .006 (p = .05 / 8 comparisons).

#### Environment

Fifty participants completed the task on a desktop and 144 individuals completed the study on a laptop device. Eight participants reported completing the task while at work and one completed it on a university campus, and all other individuals (n = 185) completed the study in their home environment. Eleven participants experienced some form of audio or visual sensory distraction during the completion of the study. These distractions were largely mild; examples included a dog barking, someone knocking at the door, and the participant’s phone ringing. No participants reported substantive technical complications that would affect performance in the task.

## Discussion

In contrast to established laboratory findings [16, 23, 25, 26, 55–59], data from our online study revealed that memory was comparable in young adults who experienced a brief period of awake quiescence or sensory processing after learning. Specifically, no benefit of post-encoding eyes-closed quiet rest (minimal sensory processing and task engagement) was observed in visual detail memory for photos of everyday items when our procedure was completed in an everyday setting. This finding was observed despite the application of a memory paradigm that has been shown to be sensitive to quiescence in the laboratory [25]. We consider explanations for this null finding and provide a set of recommendations for future research exploring the translation of rest effects in memory from the laboratory to everyday life, where impact could be achieved especially in applied settings.

Can the null finding in our study be explained by random between-group differences? Our data suggest this possibility is unlikely; the awake quiescence and perceptual task groups were matched in their age and gender distribution, as well as their visual acuity. Similarly, groups were matched in their self-reported ratings of focus, mood, sleepiness, and fatigue, both before and after their allocated delay condition. Moreover, across these measures, participants’ responses suggested that they were functioning at a level that was sufficient for the requirements of the study, for example, pre-encoding sleepiness ratings broadly corresponded to *“functioning at high levels*, *but not at peak; able to concentrate”*.

Similarly, groups were matched in encoding phase performance: we observed no significant differences in the number of missed trials or the time that participants took to respond to presented stimuli. Importantly, encoding phase data were comparable to laboratory findings [25] and high trial response rates (>98% in both groups) demonstrate that participants were engaged in the requirements of the encoding phase (judgment-making task) despite the current procedure being delivered online. While other factors, for example, depth of encoding [60], cannot be established, our findings indicate that participants in both groups received a similar experience during the encoding of photos of everyday items.

Data from the testing phase of our procedure revealed that participants’ memory for the gist and fine details of encoded photos was comparable between individuals who rested and those who played the spot-the-difference game for 10 minutes after encoding. Similarly, response times for target, lure, and foil items were comparable across groups. Standard recognition scores were close to ceiling in those who rested or completed the spot-the-difference game. Because this recognition measure reflects participants ability to accept “old” targets and reject “new” foils, this reinforces our encoding phase data and indicates that both groups successfully acquired visual memories for the presented items. It further indicates that participants in both groups experienced minimal forgetting of these visual memories (or at least their gist representations) over the course of their allocated 10-minute delay condition. This is in keeping with existing laboratory data [40, 41, 52, 61], including where comparable–and high–performance in the standard recognition measure has been reported following rest and task engagement in young adults [25].

Crucial to the current study, we observed no significant effect of delay condition in LDI scores, which reflect a participant’s ability to successfully discriminate between similar lures and identical target items. A benefit of rest in laboratory research has been reported in this measure [25], where it was proposed that quiescence provides a state of reduced sensory processing that crystallises the fine details of new visual memories through encouragement of opportunistic consolidation processes like neural replay [2–5]. While LDI scores in the perceptual task group resonate with those observed in laboratory research, numerically, LDI scores in the quiescence group are lower and demonstrate greater variability across participants [25]. Given near ceiling performance in the standard recognition measure, the lack of difference in the LDI measure cannot be explained by increased forgetting of encoded items negating the benefit of rest in participants who experienced awake quiescence. Similarly, the null finding in LDI scores is unlikely to be explained by a lack of an interference effect generated from sensory processing in the perceptual task delay condition. Participants engaged sufficiently with the demands of the spot-the-difference game as demonstrated by the number of trials responded to and proportion of correct responses, which resonates with laboratory findings where interference effects have been observed when using the same or similar perceptual task as the current study [16, 17, 19–21, 23, 25, 49, 55, 57]. We do however acknowledge that some findings are mixed [e.g., 56, 62, 63]. Furthermore, demonstrable engagement with the requirements of the spot-the-difference task does not necessarily indicate that a participant maintained a fully engaged state (of sustained attention and sensory processing) that was detrimental to consolidation. It is possible that the degree of task engagement, performance (e.g., finding two differences before the end of a trial), and other factors may have resulted in participants experiencing at least brief entry into rest-like states that were conducive to consolidation. This possibility is supported by recent neuroimaging evidence demonstrating that the brief experiencing of certain EEG microstates during post-encoding activity positively predicts subsequent memory [64]. This possibility may account for individual differences in memory performance following the filled delay though it is unlikely to account for comparable memory between our filled and unfilled delay conditions given that interference effects have been reported in related laboratory-based research [25].

Instead, it is more likely that the null finding in LDI scores can be explained by a lack of a rest effect. Specifically, because of the online nature of our study design, it is possible that participants were challenged in their ability to engage in—and maintain—a state of quiescence that was conducive to opportunistic consolidation. Indeed, our data indicate that consolidation must have occurred to a reasonable level as participants demonstrated little forgetting of encoded targets at the level of gist representations (reflected in near ceiling standard recognition scores). Still, it remains possible that consolidation was not encouraged sufficiently to support the retention of the fine details of all encoded stimuli. This explanation is supported by subjective post-experimental reports, where participants in the quiescence and perceptual task groups rated their allocated delay conditions as being comparably restful. Similarly, (i) focus and fatigue ratings were comparable between groups and did not change significantly from pre- to post-delay condition measurements, and (ii) while sleepiness increased over the course of the study, this change was comparable in both groups.

Furthermore, a portion of individuals in the quiescence group reported challenges in maintaining a state of quiescence. This included distractions in their environment (e.g., door bell ringing or someone walking into the room), not being experienced in maintaining a restful state, and being distracted by internal thoughts and feelings. Poor adherence to the requirements of their delay condition may also have affected participants’ performance. Indeed, most individuals in the quiescence group reported not keeping their eyes closed or briefly falling asleep. Such task adherence issues were not apparent for those in the perceptual task group, as demonstrated by performance in the spot-the-difference game. Given the online and remote nature of our study and the fact that participants were remunerated for their participation, it is possible, and indeed plausible, that issues surrounding task adherence are likely to be underreported.

These data are compounded by participants’ reports and ratings surrounding their mental activities during their allocated delay condition. In contrast to published findings [e.g., 25, 49, 55, 65], participants in our awake quiescence and perceptual task conditions reported similar mental activities and categories of thought, which were dominated by visual imagery and inner language. Similarly, we found no between-group difference in ruminative thoughts during their allocated delay condition, and the level of rumination was unrelated to memory performance. Mental activities pertaining to the encoded items were relatively uncommon across both groups, which is unsurprising given that they were unaware that they would complete a delayed recognition test. Still, given the unfilled nature of the quiescence delay, it is not unusual to expect greater spontaneous and intentional thought pertaining to encoded items in those who experienced this condition. While existing findings have shown such thoughts to not be largely predictive of rest effects in memory [30, 49, 55], reports of similar thoughts in the current study may indicate that participants in both groups experienced similar mental states that were comparably conducive for consolidation.

Together, our findings tentatively indicate that participants in the awake quiescence and perceptual task groups experienced similar cognitive and mental demands during their allocated delay conditions. It may be for this reason that no benefit of quiet rest was observed in LDI scores, because, in large, participants in the quiescence group did not experience a state of minimal sensory processing and task engagement that would be required to not only consolidate gist representations, but the fine details of encoded visual memories. Further to this, it is possible that the observed variability in LDI scores may reflect varying degrees of engagement and maintenance of a state of quiescence.

What are the consequences of our null findings? While research surrounding consolidation has traditionally investigated the contribution of sleep, promotion of awake consolidation to support memory retention could have a significant impact in applied settings [32]. Such impact could be felt, for example, in education and eyewitness testimony settings, as well as broader benefits in those with and without memory disorders, where more striking effects are reported [22, 28]. Indeed, restful mindfulness and meditation states have been proposed to offer potential to support learning [33] and engagement in targeted rest periods is suggested to have great potential in sporting settings [34]. However, the outcomes of the current study indicate that the memory benefit of quiescence in (at least) visual detail memory is not straightforward to achieve.

Based on the above, we raise three research questions that require further investigation to establish whether the memory benefit of awake quiescence can translate from the laboratory to everyday life.

First, what are the specific environmental and individual conditions that are conducive and detrimental to awake consolidation? Despite extensive evidence demonstrating that quiet rest is beneficial to memory; the exact mechanisms underpinning this effect remain poorly characterised. Several potential mechanisms have been proposed, including sustained attention and task engagement [23, 25], a reduction in sensory processing [20, 21] and rich autobiographical thinking activities [49, 65] that would otherwise interfere, as well as more intentional mnemonic strategies including active rehearsal [63]. However, the exact factor, or, more probable, combination of factors, that underpin the rest effect remain poorly understood. For the memory benefit of quiescence to be translated to everyday life, further characterisation of the neurocognitive underpinnings of the effect is required.

Second, what are the barriers to achieving a state of quiescence in everyday life and can such challenges be managed to promote consolidation? Our study raises important issues surrounding participants ability to engage with and maintain a state of quiescence in everyday settings. For the memory benefit of rest to be translated to applied settings, it must be easily achievable. Should complex interventions be required to promote memory consolidation for each set of new memories, adherence will be poor irrespective of the possible benefit. Thus, it is important to understand the challenges that an individual may face when engaging in a brief period of quiet rest in their everyday setting. For example, what are the most likely factors to interfere with an individual’s ability to experience rest in their everyday life and are their mechanisms through which they can be managed? Similarly, could training in what constitutes as quiescence or mindfulness and meditation help participants engage in consolidation-conducive practices. Formal exploration of such research questions can provide important insights into the factors that will limit the ability of rest to translate to everyday life for those with and without memory disorders.

Third, is it possible that knowledge of the memory benefit of awake quiescence may encourage individuals to engage in and maintain a consolidation-supporting state? Research examining awake consolidation and the benefit of quiescence has largely utilised incidental encoding and surprised delayed recall or recognition tests to reduce the likelihood of conscious memory processes (e.g., intentional rehearsal). While incidental encoding is typical of everyday memory functioning, this methodological approach means that participants are naïve to the benefit of engaging in awake quiescence and negative effect of task engagement and sensory processing after learning, at least until being debriefed. While appropriate for the laboratory, a lack of knowledge of the benefits of quiescence may result in poor engagement and maintenance of mental and physical state that is conducive to consolidation. Given that intentional encoding of episodic information is beneficial for retention complex episodic information [66], intentional encoding coupled post-encoding quiescence and retrieval [67] provide optimal conditions for awake consolidation.

Together, these three research questions can act as a set of recommendations for future studies to maximise the possibility of observing a memory benefit of post-encoding awake quiescence in applied settings.

Despite no benefit of awake quiescence being observed, it is reassuring that we experienced no notable issues in the online delivery of our memory paradigm: performance levels and trial response rates were broadly in keeping with laboratory research and suggest successful engagement with task requirements. Furthermore, a greater number of items were encoded and subsequently probed in the testing phase of the current study relative to exiting laboratory work [25] though the number of items in the current study reflect that used in non-consolidation focused research [31, 40, 41]. This was done in attempt to improve test sensitivity though may have worked to our detriment, where the increased encoding load may have pressured consolidation to the degree where no benefit of rest could be observed, or the effect on each item was too minimal to be detected.

## Conclusion

In summary, the outcomes of the current study conflict with existing findings demonstrating a memory benefit of awake quiescence–commonly reported under lab conditions–and indicate that such an effect may be challenging to observe in everyday life, at least in the domain of visual detail memory. There is value in future research examining the specific requirements of the laboratory and everyday life environments that are needed to successfully translate the memory benefit of awake quiescence into applied settings.
